# A study based on functional near-infrared spectroscopy: Cortical responses to music interventions in patients with myofascial pain syndrome

**DOI:** 10.3389/fnhum.2023.1119098

**Published:** 2023-01-27

**Authors:** Jiayue Zhang, Ping Shi, Jiahao Du, Hongliu Yu

**Affiliations:** Institute of Rehabilitation Engineering and Technology, University of Shanghai for Science and Technology, Shanghai, China

**Keywords:** functional near-infrared spectroscopy, music intervention, myofascial pain syndrome, chronic pain, cortical response

## Abstract

**Object:**

This study measured cerebral blood oxygen changes in patients with myofascial pain syndrome (MPS) using functional near-infrared spectroscopy (fNIRS). The aim was to investigate the effect of music intervention on pain relief in MPS patients.

**Materials and methods:**

A total of 15 patients with MPS participated in this study. A self-controlled block task design was used to collect the oxy-hemoglobin ([HbO_2_]) and deoxy-hemoglobin ([HbR]) concentrations in the prefrontal cortex (PFC) and motor cortex using fNIRS. The cerebral cortex response and channel connectivity were further analyzed. In the experiment, the therapist was asked to apply compression of 3–4 kg/cm^2^ vertically using the thumb to induce pain. Soothing synthetic music with frequencies of 8–150 Hz and 50–70 dB was used as the audio for the music intervention.

**Result:**

Compared to the group without music intervention, the activation of brain regions showed a decreasing trend in the group with music intervention under the onset of pain. The results of paired *t*-tests showed that nine of the data were significantly different (*p* < 0.05). It was also found that with music intervention, inter-channel connectivity was diminished. Besides, their dorsolateral prefrontal cortex (dlPFC) was significantly correlated with the anterior prefrontal cortex (aPFC) for pain response (r = 0.82), and weakly correlated with the premotor cortex (r = 0.40).

**Conclusion:**

This study combines objective assessment indicators and subjective scale assessments to demonstrate that appropriate music interventions can be effective in helping to relieve pain to some extent. The analgesic mechanisms between relevant brain regions under music intervention were explored in depth. New insights into effective analgesic methods and quantitative assessment of pain conditions are presented.

## 1. Introduction

Chronic pain is a disease with complex causes, high prevalence, and plagues people of all ages. It lasts for no less than 3 months and recurrent episodes (Benoliel et al., 2019). According to statistics, the number of chronic pain patients in China has exceeded 300 million, while 10–20 million people are added every year, Overall, there is a trend of rapid growth and youth (Fan, 2020). Among them, myofascial pain syndrome (MPS) is a common form of chronic musculoskeletal pain. The disease has a complex etiology and occurs in multiple parts of the body. It is also characterized by a fixed pressure point (Wang and Pan, 2022). In the general population, the lifetime prevalence of MPS is as high as 85% (Galasso et al., 2020). The daily life of such patients can be seriously affected by pain, which also brings heavy physical, psychological and economic burdens to the family. Nowadays, how to help patients with pain has gradually become a medical issue of attention. In the present study, we used patients with this disorder as the primary objects.

Medications have always been the primary choice for chronic pain management. However, considering the adverse effects of opioids, many complementary therapies are gradually emerging (Agoston and Sieberg, 2016). In view of the causes of pain are complex and are closely related to the patient’s own emotional and psychological problems. Previous studies have also shown that, among many other treatments, music intervention is a non-invasive and highly actionable approach to reduce pain in patients with pain (Hole et al., 2015; Choi et al., 2018). So the advantages of musical interventions are gradually emerging. Qi (2004) found that appropriate music can attenuate sympathetic activity and enhance parasympathetic activity in the body, suppressing anxiety situations. Music can also help patients stabilize their physiological rhythms, relax their bodies and minds, and relieve pain symptoms. In addition, long-term music intervention can stimulate melatonin secretion, which can effectively relieve patients’ insomnia and other symptoms that accompany pain (Shao and Wang, 2009; Shi et al., 2022).

However, the current evaluation of pain mostly relies on the use of subjective scale scores, the amount of analgesics used (Peng et al., 2018b), and the duration of pain tolerance (Cheng et al., 2017), which always lacks an objective way of assessment. With the introduction of electroencephalogram (EEG), functional magnetic resonance imaging (fMRI), and functional near-infrared spectroscopy (fNIRS), a new way of studying brain function has been offered. They are increasingly being recognized as a potential diagnostic and predictive tool for the treatment of patients with chronic pain (Davis and Seminowicz, 2017). EEG is often used in previous studies (Meagher and Albu, 2016; Taesler and Rose, 2016) to assess the relationship between pain and brain function. Nowadays, more scholars choose to use fNIRS for pain study, mainly because of its high temporal resolution, small size, portability, ease of wear, and insensitivity to motion artifacts. It can be used to objectively evaluate the level of brain response by measuring changes in blood flow in the cerebral cortex and thus it can be used to study the brain’s response to nociception (Hu et al., 2021). It also has great potential for objective pain assessment in clinical settings and has been valued. Peng et al. (2018a) used fNIRS to measure the prefrontal cortex in 14 participants, demonstrating the feasibility of fNIRS for pain measurement. Gentile et al. (2020) recruited 38 fibromuscular patients and 21 healthy individuals. By observing changes in the motor cortex, complex mechanisms of interaction between networks of pain control and motor function were explored.

Although fNIRS has been widely used in pain study, few scholars have used fNIRS to assess the pain relief effects of music interventions. Therefore, this study will use the fNIRS device for data collection to explore the observed cortical response to pain in participants with and without music interventions. Indicators such as blood oxygen signals, brain activation levels, and brain networks will be further analyzed to objectively assess the effect of music intervention on pain.

## 2. Materials and methods

### 2.1. Participants

A total of 15 participants (nine females, six males, Mage = 37.87 ± 15.34 years) were recruited for this study. Referring to the Simons 1990 clinical criteria for the diagnosis of myofascial pain syndrome (Patrick, 2000), patients eligible for this type of chronic pain were included in the study. Exclusion criteria included: (1) unconsciousness, severe cognitive impairment, and visual or hearing impairment; (2) previous history of psychiatric disorders; (3) skin breakdown at the site of compression pain; (4) inability or refusal to cooperate; and (5) other patients who did not belong to myofascial pain syndrome. The study was reviewed and approved by the Medical Ethics Committee of Huadong Hospital Affiliated to Fudan University (No. 2021K104-F221). All participants were informed and agreed to cooperate with the study, and the basic information of the participants is shown in Table 1.

**TABLE 1:** Basic information of subjects.

	Gender	Age	Area of pain
S1	M	22	Shoulder
S2	F	44	Hip
S3	F	26	Shoulder
S4	F	23	Neck
S5	M	67	Back
S6	M	25	Back
S7	F	50	Back
S8	F	38	Back
S9	F	23	Neck
S10	F	59	Shoulder
S11	F	58	Shoulder
S12	F	48	Shoulder
S13	M	25	Neck
S14	M	27	Shoulder
S15	M	33	Neck

### 2.2. Task and procedure

The experiments were conducted in a quiet room, and the participants were familiarized with the experimental procedure in advance. During the experiments, the participants were asked to sit in a chair in a comfortable position and to remain as quiet as possible, avoiding large body and head movements to reduce interference with the fNIRS signal acquisition.

**FIGURE 1:**
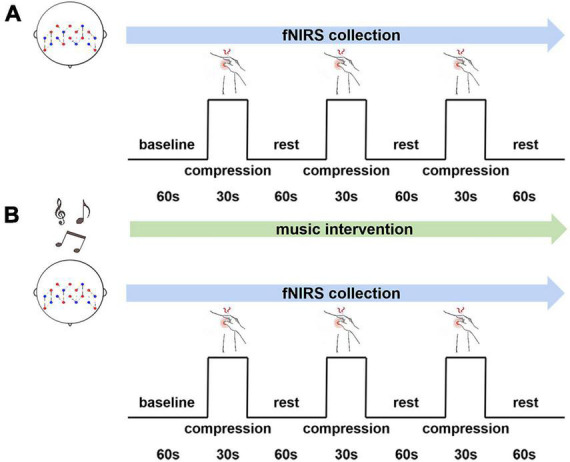
An experimental paradigm for music intervention. **(A)** Without music intervention. **(B)** With music intervention.

A self-comparison type of experiment was used in our study. A block task was designed including three groups of compression pain stimuli and three groups of rest. The experimental paradigm is shown in Figure 1. The baseline time as well as the rest period required participants to relax with their eyes closed, no additional task was required. Headphones were worn at all times during the experiment. The experimental tasks were identical for both groups and the fNIRS device was used for data collection throughout. Each participant completed two experiments according to the experimental paradigm, one without and one with music interventions. There is a 15 min break between them.

According to the preliminary research (Kayashima et al., 2017; Martin-Saavedra et al., 2018) we found that there was no significant correlation between the music type and the effectiveness of the music intervention. Besides, preference for music may not influence it either. Therefore, we chose a unified music for this study. Considering that 60–85 dB is the optimal sound threshold range for the human ear (White, 2000), also, music at 8–13 Hz can stimulate alpha brain waves, thus helping consciousness to relax (Deng et al., 2013). Also the low frequency signal of 16–150 Hz in music can help to relieve pain, reduce stress, and so forth (Bai et al., 2010). Therefore, together with white noise and brainwave induction techniques, soothing synthetic music with frequencies of 8–150 Hz and 50–70 dB was used for the music intervention. To avoid possible persistent effects even after music pauses, experiments without music interventions were conducted first, followed by music.

According to the characteristics of myofascial pain syndrome, pressure on the painful area triggers regional pain. An experienced therapist was asked to apply compression to the area of pain to induce pain (see Table 1 for details), and the participants were asked for visual analogue scales (VAS) for pain with pressure (Ye et al., 2021). The therapist was trained before the experiment and was subsequently asked to perform 10 compression tests using a pressure transducer. The compression force was measured to be 3.52 ± 0.221 kg/cm^2^, and each compression force was relatively even. The study (Tong et al., 2022) proved that 3–4 kg/cm^2^ could induce pain and therefore could meet the experimental requirements.

### 2.3. Imaging acquisition

**FIGURE 2:**
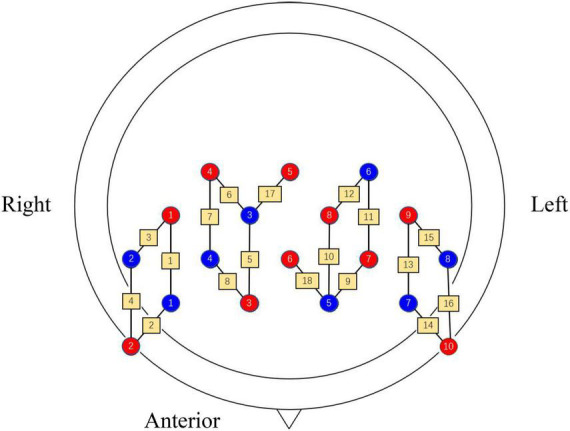
Arrangement of each channel, where red indicates the light source, blue indicates the receiver source and yellow pathways indicate the channels.

The Brite 24 (Netherlands) system was used to acquire data with light sources of 760 and 850 nm. The device consists of 10 light sources and eight receiver sources, and the distance between them is 30 mm by default. To synchronize the recording of noise from the brain surface to remove interference, the channels can be set up for short interval pathways with a distance of 15 mm between the light source and the receiving source (such as CH17 and CH18). fNIRS channels are set as shown in Figure 2. The acquisition rate of the device was set to 10 Hz to capture the changes of oxyhemoglobin ([HbO_2_]) and deoxyhemoglobin ([HbR]) concentrations in the participant’s brain in real-time. When the brain tissue is excited, the trend of increasing [HbO_2_] concentration in the blood vessels of the local brain activation area is more significant (Iso et al., 2021). Therefore, the trend of [HbO_2_] concentration changes was selected as the main observable index.

The “pain matrix” theory was proposed by Melzack in the 1980s, based on the observation of brain regions by pain imaging. It covers several regions of the cerebral cortex, and brain activation is considered to be more obvious and objective evidence that an individual is experiencing pain (Legrain et al., 2011). The key brain region for assessing pain is the prefrontal cortex (PFC), which is the supreme central system for nociceptive coding and is mainly responsible for integrating sensory and emotional information (Baliki et al., 2008). Therefore, in the present study, it was used as the primary concerned brain region. Nine channels were placed in each of the left and right brain as measurement areas, and both areas were placed symmetrically. BA6, BA9, BA10, and BA46 were used as regions of interest (ROI) according to the Brodmann area (see Table 2).

**TABLE 2:** Corresponding brain regions, Brodmann, channels and coordinates.

Brain regions	Brodmann	Channels	MNI coordinates		
			**X**	**Y**	**Z**
PMA and SMA	BA6	CH3	63	16.333	11.667
		CH15	−61	15.333	15.667
aPFC	BA10	CH5	27.667	67	18
		CH9	−36.667	64	0
		CH10	−15.333	71.667	15
		CH18	−10	72	14.667
dlPFC	BA9	CH17	14	59.667	38
	BA46	CH8	45.333	57.667	6.333

### 2.4. Data analysis

To remove the unavoidable noise interference, including baseline drift, physiological signal interference, motion artifacts, and industrial frequency noise, during data acquisition, the data processing is divided into the following steps. First, the acquired fNIRS data are de-drifted to remove motion artifacts, such as head movement signals (Cui et al., 2010). The processed data is then passed through a bandpass filter of 0.01–0.1 Hz to filter out the general noise. Finally, the shallow noise recorded by the short interval pathway was removed from the total signal by noise regression to complete the pre-processing operation.

We used a generalized linear model (GLM) for the analysis of brain activity under different experimental conditions to estimate the activation of brain regions (Schroeter et al., 2004), as shown in formula (1). Where y denotes the observed data, X denotes the desired reference value, β denotes the parameter value to be estimated, and ε denotes the residuals. Thus, under the guidance of this model, the individual hemodynamic response index can be calculated by estimating the sum of each channel to calculate individual hemodynamic response indices.


(1) y=X⁢β+ε

### 2.5. Statistical analysis

The NIRS-KIT software was used to process the values obtained for the activation in the participant’s brain. Referring to the GLM model, values for periods of compression pain stimulation and rest periods were obtained by interpolation and smoothing operations on the human brain. Statistical analysis was performed using SPSS 26.0 software. The normality of the data was verified using the Shapiro-Wilk test, which conforms to a normal distribution. Subsequently, to test for differences in activation between channels, a paired *t*-test was performed on values with and without the music intervention. *p* < 0.05 indicates a significant difference. In addition, the Pearson correlation coefficient was used for functional connectivity analysis (FC). [HbO_2_] was calculated for each participant under both conditions, and individual FC was obtained from each channel time series. The mean value was taken to obtain the group mean FC, and an 18 × 18 group level matrix was generated.

## 3. Results

### 3.1. Trends of [HbO_2_] concentration

**FIGURE 3:**
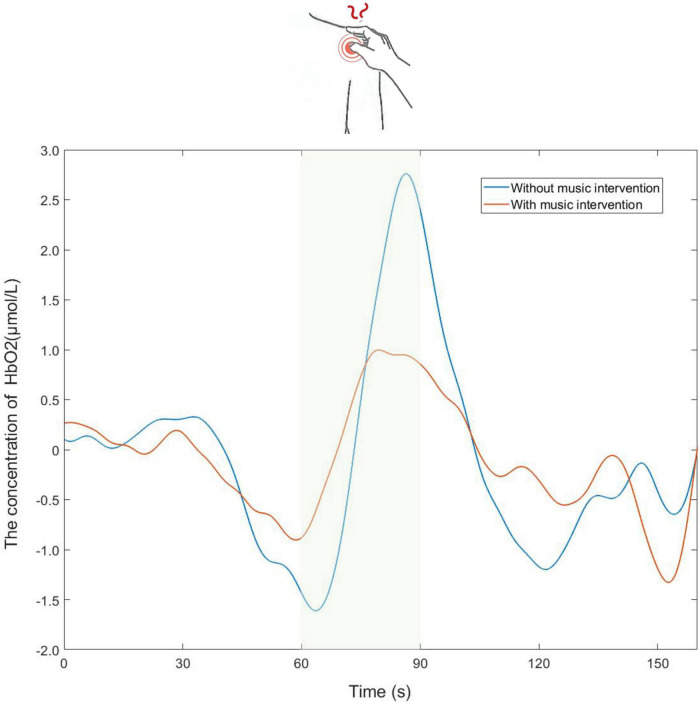
Partial time series plot of the oxy-hemoglobin ([HbO_2_]) concentration response of the channels covered by the regions of interest (ROI) brain region.

Shown in Figure 3 are the trends of [HbO_2_] concentration changes in the channels covered by the ROI brain region during the experiment. It can be seen from the figure that during the resting state, [HbO2] concentrations showed sinusoidal dynamic changes (Han et al., 2017). However, there was a clear upward trend in the presence of pain-inducing tasks (shaded in green), while activation occurred in the brain areas observed. The peak in [HbO_2_] concentration occurred during the pain-induced task period without music intervention. Meanwhile, the overall trend of change with music intervention was flat compared to the trend without music intervention.

### 3.2. Activation of brain regions

**FIGURE 4:**
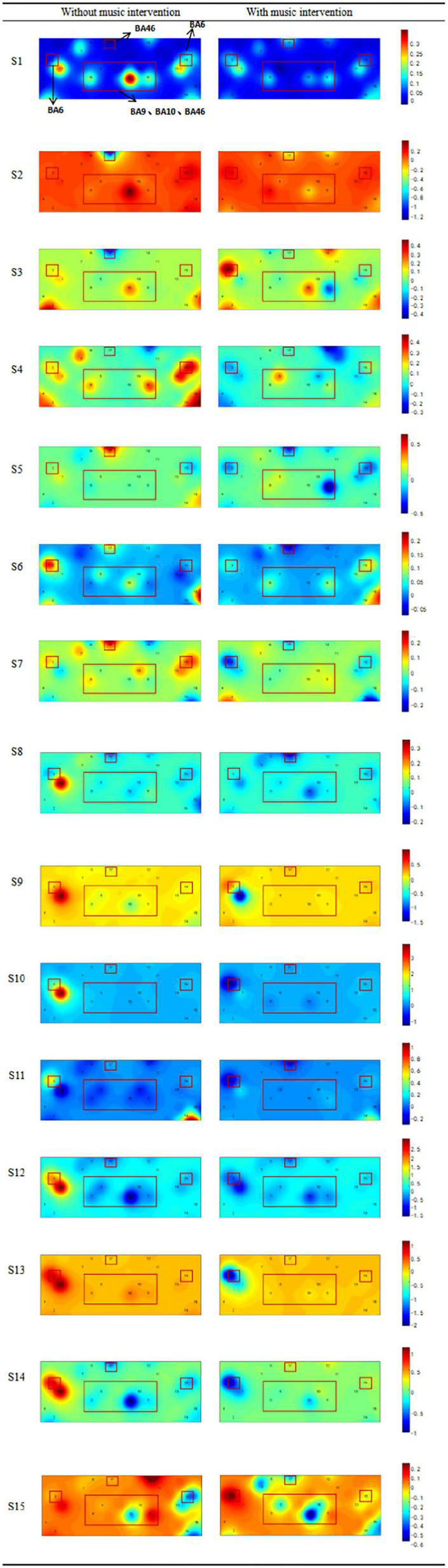
Comparison of activation levels in the prefrontal cortex (BA9, BA10, BA46) and motor area (BA6) with or without music intervention. The color from blue to red indicates a continuous increase in brain activation. The regions of interest (ROI) brain regions mentioned above that are associated with pain have been framed, including the prefrontal cortex and motor cortex.

The brain areas examined by the device also showed different degrees and ranges of activation, as shown in Figure 4. It shows the brain activation of participants in both conditions, with different colors indicating the activation of the brain channels. It was found that the brain areas monitored during the music intervention tended to have a reduced activation range, with a general decrease in activation and a certain relief of brain tissue excitation.

**FIGURE 5:**
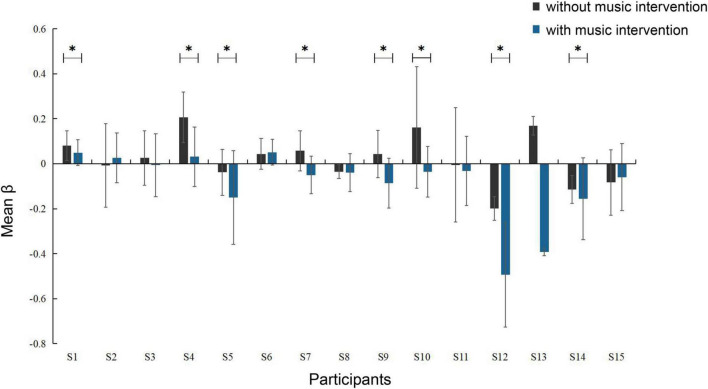
Degree of activation of brain areas in conditions with and without music intervention (*p < 0.05). The error bars indicate the standard error of the mean.

The activation intensity was calculated separately according to the activation level of the channels in the task, with higher values indicating stronger activation. A comparison of the activation intensity of each ROI for all participants with and without the music intervention is shown in Table 3. It can be seen that the group with music intervention was significantly lower than the other group. The channels in the ROI were selected to compare the brain activation levels of each participant in the condition with and without the music intervention, as shown in Figure 5. As can be seen from the figure, the mean activation levels of the participants under the music intervention were generally lower than those without the music intervention. Nine of the participants experienced significant pain relief (*p* < 0.05).

**TABLE 3:** Comparison of activation intensity between the two groups.

Group	*n*	β ($\bar{x}\pm s$)			
		**BA6**	**BA9**	**BA10**	**BA46**
Without music intervention	15	0.152 ± 0.191	0.244 ± 0.498	0.021 ± 0.181	0.114 ± 0.170
With music intervention	15	−0.153 ± 0.340	−0.175 ± 0.292	−0.069 ± 0.157	−0.050 ± 0.144
t	–	2.504	2.301	2.178	2.160
*P*	–	0.025*	0.037*	0.047*	0.049*

*p 0.05.

### 3.3. Channel connectivity analysis

**FIGURE 6:**
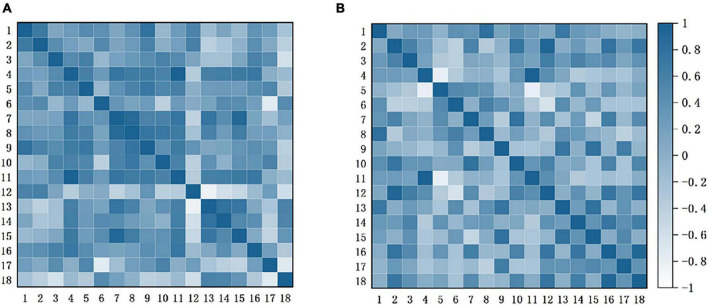
Functional connection matrix for each channel with and without music intervention. **(A)** Without music intervention. **(B)** With music intervention. It presents the connectivity matrix heat map for the two conditions with and without music intervention, respectively. In this case, each pixel value in the 18 ×18 matrices corresponds to a value of the Pearson correlation coefficient, which is used to express the correlation between the channels.

Figure 6 shows the FC between the channels. Each pixel value in the matrix corresponds to a Pearson correlation coefficient value, and the Pearson correlation coefficient indicates the correlation between the measured channels. As can be seen that the correlations between the channels differed significantly in both conditions. In the condition without music intervention, strong correlations existed between the channels, especially for CH3-5 and CH7-11. In the other condition, the correlations between the channels generally diminished.

### 3.4. Correlation of ROI brain regions

**FIGURE 7:**
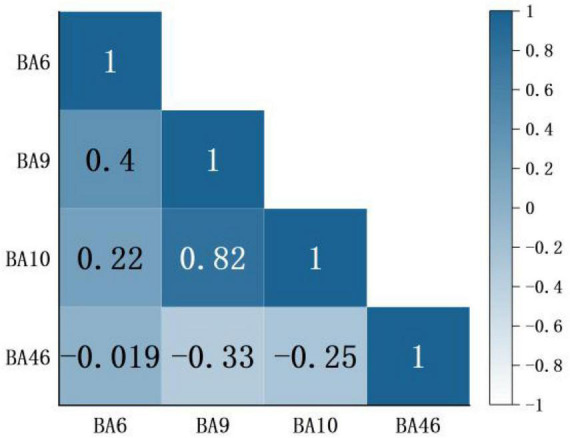
A correlation of regions of interest (ROI) brain region in the condition of music intervention. Red represents a positive correlation, blue represents a negative correlation, and darker color means a more significant correlation. The number indicates the correlation coefficient r. Usually |*r*|≥0.7 defined as high correlation, 0.3 < |*r*| < 0.5 defined as low correlation, and |*r*|≤0.3 defined as no correlation.

Based on channel connectivity, ROI was selected, and Pearson analysis was continued on the situation regarding [HbO_2_] concentration under music intervention. These results are shown in Figure 7. Among them, BA9 and BA10 have high linear correlation (*p* < 0.05). BA6 and BA9 have low linear correlation (*p* < 0.05), and the correlation between the remaining brain regions is not strong.

### 3.5. VAS score

**FIGURE 8:**
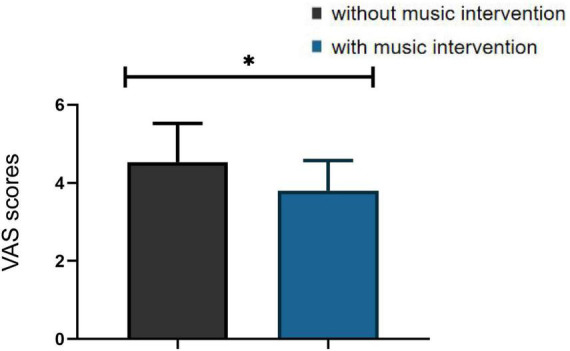
Visual analogue scales (VAS) scores in the condition with and without musical intervention (*p < 0.05).

According to the participants’ subjective self-report, they were distracted and their pain condition was partially relieved in the presence of the music. They were also asked to perform subjective pain scores using the VAS. As the Figure 8 showed that those in the condition with music intervention were significantly lower than those in the condition without music intervention, and there was a significant difference between the two groups (*p* < 0.05). The association between β values and VAS scores for the assessment of pain was further analyzed using chi-square tests. The β values represent the objective reflection of the fNIRS signal on pain changes and the VAS score represents the subjective perception of the participants. The results of the analysis revealed significant correlations between values and VAS scores for the assessment of pain (χ^2^ = 5.104, *p* < 0.05).

## 4. Discussion

Music therapy has been widely used in clinical practice and has been shown to have a positive effect on the treatment of pain. Psychology believes that music can be used as a therapeutic tool, mainly due to its mood-boosting, anxiety-reducing, and mood-improving effects (Bernatzky et al., 2011). Therefore, it can reduce the patient’s perception of pain and regulate the psychological state, thus relieving pain. This is also the general opinion of music therapy. It has also been found that there is a close connection between music and functions of the brain (Luo et al., 2022). Based on the changes in blood oxygen concentration detected by the fNIRS device in response to brain function, we objectively verified that soothing music can alleviate pain conditions to some extent.

### 4.1. The fNIRS signal may reflect changes in pain conditions

Quantitative analysis showed that changes in the [HbO_2_] signal acquired by fNIRS contributed 73–79% to the measured changes in total hemoglobin concentration (Hu et al., 2021). Therefore, this study was based on the [HbO_2_] signal from fNIRS for further analysis. The analysis of β values and VAS scores according to this paper in 2.5 revealed a significant correlation between the results of objective and subjective assessments, which can also corroborate the idea that fNIRS signals can reflect changes in pain conditions.

It is known from previous fNIRS studies that using the average values of task blocks in the statsitic analysis is a common method (Yang et al., 2020; Xia et al., 2022). The trend of the change in [HbO_2_] concentration after taking the mean value for the three tasks is shown in Figure 3. In-depth, it was found that when the same stimulus was applied to the participant multiple times, it caused a habituation effect, resulting in a slight weakening of the signal (Yucel et al., 2015). Thus the habituation effect may lead to slight deviations in the trend of change. Meanwhile, it is evident from Figure 3 that the trend of [HbO_2_] concentration change with music intervention was flatter and the peak value was significantly lower than that without music intervention. It is thus hypothesized that the music intervention has some effect on analgesia.

### 4.2. Brain area response to music intervention

It is known from relevant studies that patients with pain are more prone to negative emotions such as anxiety and depression. There is often a complex relationship between pain and emotional problems, and they affect each other (Fishbain et al., 1997; Mccracken et al., 1999). And music can help patients regulate their emotions, which will have an impact on the activation status of PFC (Peretz, 2001). Therefore, the study used the PFC as the main observed brain region to obtain abundant pain-related information and implement further in-depth studies.

The PFC is connected to several other known pain-related brain regions, such as the insular cortex and parietal cortex, which synergistically modulate pain perception processes. Several pain-related studies, including cold stimulation (Pourshoghi et al., 2016; Sharini et al., 2019), thermal stimulation (Yennu et al., 2016), mechanical pressure stimulation of the fingers (Chih-Hung et al., 2013) and lower back (Vrana et al., 2016), have confirmed that PFC activation is positively correlated with increased pain. In our study, when stimulation occurred, the PFC brain region became excited, the [HbO_2_] concentration increased, and activation of the brain region occurred. Thus confirming that activation of the PFC brain region occurs accordingly during pain onset. Depending on their location in the prefrontal cortex, the functions they are responsible for vary considerably and represent different meanings.

Bsliki (Baliki et al., 2011) demonstrated in their findings that the PFC is usually activated during pain and that the activation is positively correlated with the enhancement of pain perception. The dlPFC is primarily involved in the capacity for attention, working memory, and motivational execution. Gustin et al. (2010) found that the intensity of pain experienced by patients with all types of pain was significantly correlated with the magnitude of dlPFC activation. Other related studies have shown that activation of the dlPFC is associated with midbrain excitation. Activation leads to the release of opioid peptides, which in turn relieves pain (Li et al., 2022). The topography of activation of brain regions in Figure 4 provides changes in the location and extent of activation of the PFC with and without music intervention. From our study, it is clear that after the music intervention, the prefrontal brain regions showed different extents of diminished response to pain and reduced excitatory areas of the brain, which is consistent with the findings of previous studies (Peretz, 2001). The ventromedial prefrontal cortex (vmPFC) brain region is also part of the PFC and this brain region is mainly associated with the management of negative emotions (Qin, 2014). Its activation was diminished in the presence of the music intervention and the relief of negative emotions was also associated with its reduced activity. Therefore, it can be inferred that the music intervention helped the participants to alleviate negative emotions such as anxiety (Hirschfeld, 2011).

In addition to PFC areas, this study also found a trend of decreased activation in related motor areas. Based on the corresponding functions of brain regions, it is known that SMA mainly controls the proximal and trunk muscles of the body. The activation of this region is associated with pain avoidance and is a conditioned reflex in the body. In the music intervention condition, the participants’ muscle tension decreased, which had a relaxing effect on muscle tone and reduced the participants’ subconscious avoidance response to pain (Guétin et al., 2005). Changes in motor areas in the conditions with and without music intervention are also shown in Figure 4. The general decrease in activation in the music condition is consistent with the findings of previous studies that motor areas also reflect analgesia (Zhang et al., 2001). Thus, it can be inferred that a decrease in the activation of the motor area could also indicate the relief of the pain condition.

### 4.3. Mechanisms and effects of analgesia with music intervention

Several studies have shown that nociceptive information is transmitted upward through the spinal cord to the brain, into relevant brain regions such as the thalamus, amygdala, anterior cingulate gyrus, and insula. Through structures connected to the medial prefrontal cortex (mPFC), thus constituting nociception and pain-related emotions (Tracey and Mantyh, 2007; Apkarian et al., 2009). The analgesic effect produced by music interventions is a top-down mechanism of action. In relevant brain imaging studies, it has been shown that the dlPFC may be involved in cortical mechanisms of nociceptive modulation (Lin et al., 2009). dlPFC distracts patients from painful thoughts by coordinating with prefrontal cortical regions. It helps patients to relax by regulating their emotions and cognition, which in turn regulates pain (Jensen et al., 2012). This is the mechanism of action of music interventions for pain relief. Therefore, from the correlation of ROI in Figure 7, a significant positive correlation was found between BA9 and BA10. So dlPFC and prefrontal cortical regions act in coordination, and this study verified this idea.

In addition, some motor areas also played a role in the nociceptive experiment. This study concluded that there was also a weak positive correlation between BA6 and BA9, indicating that motor areas were also involved. This may be caused by distraction. The distraction reduces the patient’s stress avoidance response to pain, pain sensitivity is significantly reduced, and the motor area reflects the analgesic effect (Usui et al., 2020). The distraction of attention is the basic principle of pain therapies. Since human attention is finite, music interventions can achieve pain relief by partially diverting attention from unpleasant mental activities and helping patients to stop concentrating on noxious stimuli, thus reducing their perception of pain (Mobily et al., 1993).

FC has been widely used to study the interactions between brain regions, thus helping to understand the mechanisms of chronic pain. Related studies have found that pain leads to a significant enhancement of connectivity between networks related to emotion, cognitive control networks, and somatosensory-related networks (Zheng et al., 2020). The main brain region observed was the PFC associated with emotion, cognition, etc. As can be seen in Figure 6, the connectivity between the channels responsible for pain perception and pain regulation was significantly enhanced in the condition without music intervention. In contrast, the connectivity between the channels was generally reduced under music intervention. Therefore, this finding also confirms that music intervention has a relieving effect on pain.

It is clear from Figure 5 that nine participants had significant pain relief (*p* < 0.05) and several other participants had varying but not significant pain relief. This is because each person’s perception of music is slightly different, and their emotions are very subjective. Different styles of music have different effects on them, so the actual effect may not always be significant. Today, clinical studies have well-validated that music intervention is a non-invasive intervention that can help patients with pain. However, the factors associated with it are still complex and unknown (Robb et al., 2011).

## 5. Limitations

Due to the diversity of chronic pain and its complex etiology, it is controversial whether different chronic pains have the same pain response areas (Xin et al., 2012). Previous studies have also shown that pain sensitivity varies across age groups (Zhang et al., 2021). Therefore, our subsequent studies will address these issues in depth, expanding the number of participants and disease types. Add EEG and fMRI together to investigate the mechanisms by which music improves the response to pain conditions in other brain regions. In addition, the types of music will be added to explore the variability of neurophysiological responses to pain stimulation in patients using different frequency bands of music.

## 6. Conclusion

This study combined the collected cerebral blood oxygen signals with data related to functional brain activity and found that changes in [HbO_2_] concentrations in relevant brain regions can objectively reflect a person’s pain situation. It also found that appropriate music could help to distract and relieve anxiety and relief pain. This confirms the view of many previous studies on the effect of music interventions on pain relief. It was also verified that during the onset of pain, in addition to the activation of emotion-related PFC brain areas, the related motor areas are also activated, and there is a correlation between them.

## Data availability statement

The original contributions presented in this study are included in this article/supplementary material, further inquiries can be directed to the corresponding author.

## Ethics statement

The studies involving human participants were reviewed and approved by the Ethics Committee of Huadong Hospital Affiliated to Fudan University (No. 2021K104-F221). The patients/participants provided their written informed consent to participate in this study.

## Author contributions

JZ, JD, and PS designed and conducted the experiments. JZ analyzed the data and wrote the manuscript. PS and HY reviewed and modified the manuscript. All authors contributed to the article and approved the final version.

## References

[B1] Agoston A. M. Sieberg C. B. (2016). Nonpharmacologic treatment of pain. *Semin. Pediatr. Neurol.* 23 220–223. 10.1016/j.spen.2016.10.005 27989329

[B2] Apkarian A. V. Baliki M. N. Geha P. Y. (2009). Towards a theory of chronic pain. *Prog. Neurobiol.* 87 81–97. 10.1016/j.pneurobio.2008.09.018 18952143 PMC2650821

[B3] Bai J. Chen R. Lv T. Wang W. (2010). Vibroacoustic therapy and its application in medicine. *J. Hebei Med. Univ.* 40 1113–1116.

[B4] Baliki M. N. Baria A. T. Apkarian A. V. (2011). The cortical rhythms of chronic back pain. *J. Neurosci.* 31 13981–13990. 10.1523/jneurosci.1984-11.2011 21957259 PMC3214084

[B5] Baliki M. Geha P. Apkarian A. Chialvo D. (2008). Beyond feeling: Chronic pain hurts the brain, disrupting the default-mode network dynamics. *J. Neurosci.* 28 1398–1403. 10.1523/jneurosci.4123-07.2008 18256259 PMC6671589

[B6] Benoliel R. Svensson P. Evers S. Wang S. J. Barke A. Korwisi B. (2019). The IASP classification of chronic pain for ICD-11: Chronic secondary headache or orofacial pain. *Pain* 160 60–68. 10.1097/j.pain.0000000000001435 30586072

[B7] Bernatzky G. Presch M. Anderson M. Panksepp J. (2011). Emotional foundations of music as a non-pharmacological pain management tool in modern medicine. *Neurosci. Biobehav. Rev.* 35 1989–1999. 10.1016/j.neubiorev.2011.06.005 21704068

[B8] Cheng J. Jiao C. Luo Y. Cui F. (2017). Music induced happy mood suppresses the neural responses to other’s pain: Evidences from an ERP study. *Sci. Rep.* 7:13054. 10.1038/s41598-017-13386-0 29026123 PMC5638847

[B9] Chih-Hung L. Takashi S. Aiko K. Ayako K. Fukue F. Chen Y. W. (2013). Analysis for distinctive activation patterns of pain and itchy in the human brain cortex measured using near infrared spectroscopy (NIRS). *PLoS One* 8:e75360. 10.1371/journal.pone.0075360 24098378 PMC3789686

[B10] Choi S. Park S. G. Lee H. H. (2018). The analgesic effect of music on cold pressor pain responses: The influence of anxiety and attitude toward pain. *PLoS One* 13:e0201897. 10.1371/journal.pone.0201897 30080889 PMC6078312

[B11] Cui X. Bray S. Reiss A. L. (2010). Functional near infrared spectroscopy (NIRS) signal improvement based on negative correlation between oxygenated and deoxygenated hemoglobin dynamics. *Neuroimage* 49 3039–3046. 10.1016/j.neuroimage.2009.11.050 19945536 PMC2818571

[B12] Davis K. D. Seminowicz D. A. (2017). Insights for clinicians from brain imaging studies of pain. *Clin. J. Pain* 33 291–294. 10.1097/AJP.0000000000000439 27648589 PMC5340583

[B13] Deng X. Liu Q. Zhang W. (2013). Progress in the application of music therapy in pain intervention treatment. *J. Clin. Anesthesiol.* 29 1232–1234.

[B14] Fan B. (2020). *Chinese pain medicine development report.* Beijing: Tsinghua University publishing house.

[B15] Fishbain D. A. Cutler R. Rosomoff H. L. Rosomoff R. S. (1997). Chronic pain-associated depression: Antecedent or consequence of chronic pain? A review. *Clin. J. Pain* 13 116–137. 10.1097/00002508-199706000-00006 9186019

[B16] Galasso A. Urits I. An D. Nguyen D. Borchart M. Yazdi C. (2020). A comprehensive review of the treatment and management of myofascial pain syndrome. *Curr. Pain Headache Rep.* 24:43. 10.1007/s11916-020-00877-5 32594264

[B17] Gentile E. Brunetti A. Ricci K. Delussi M. Bevilacqua V. de Tommaso M. (2020). Mutual interaction between motor cortex activation and pain in fibromyalgia: EEG-fNIRS study. *PLoS One* 15:e0228158. 10.1371/journal.pone.0228158 31971993 PMC6977766

[B18] Guétin S. Coudeyre E. Picot M. C. Ginies P. Graber-Duvernay B. Ratsimba D. (2005). Effect of music therapy among hospitalized patients with chronic low back pain: A controlled, randomized trial. *Ann. Réadapt. Méd. Phys.* 48:217. 10.1016/j.annrmp.2005.02.003 15914256

[B19] Gustin S. M. Wrigley P. J. Henderson L. A. Siddall P. J. (2010). Brain circuitry underlying pain in response to imagined movement in people with spinal cord injury. *Pain* 148 438–445. 10.1016/j.pain.2009.12.001 20092946

[B20] Han Y. Yuan B. Zhang Y. Wang X. Lang W. Yan X. (2017). Role of fNIRS technology in observing the effect of needling Hegu (LI 4) on the functions of prefrontal cortex in healthy volunteers. *J. Acupunct. Tuina Sci.* 15 94–98. 10.1007/s11726-017-0982-2

[B21] Hirschfeld R. M. A. (2011). Deep brain stimulation for treatment-resistant depression. *Am. J. Psychiatry* 168 455–456. 10.1176/appi.ajp.2011.11020231 21536698

[B22] Hole J. Hirsch M. Ball E. Meads C. (2015). Music as an aid for postoperative recovery in adults: A systematic review and meta-analysis. *Lancet* 386 1659–1671. 10.1016/S0140-6736(15)60169-6 26277246

[B23] Hu X. S. Nascimento T. D. DaSilva A. F. (2021). Shedding light on pain for the clinic: A comprehensive review of using functional near-infrared spectroscopy to monitor its process in the brain. *Pain* 162 2805–2820. 10.1097/j.pain.0000000000002293 33990114 PMC8490487

[B24] Iso N. Moriuchi T. Fujiwara K. Matsuo M. Mitsunaga W. Hasegawa T. (2021). Hemodynamic signal changes during motor imagery task performance are associated with the degree of motor task learning. *Front. Hum. Neurosci.* 15:603069. 10.3389/fnhum.2021.603069 33935666 PMC8081959

[B25] Jensen K. B. Berna C. Loggia M. L. Wasan A. D. Gollub R. L. (2012). The use of functional neuroimaging to evaluate psychological and other non-pharmacological treatments for clinical pain. *Neurosci. Lett.* 520 156–164. 10.1016/j.neulet.2012.03.010 22445888 PMC3810294

[B26] Kayashima Y. Yamamuro K. Makinodan M. Nakanishi Y. Wanaka A. Kishimoto T. (2017). Effects of canon chord progression on brain activity and motivation are dependent on subjective feelings, not the chord progression per se. *Neuropsychiatr. Dis. Treat.* 13 1499–1508. 10.2147/NDT.S136815 28652751 PMC5476716

[B27] Legrain V. Iannetti G. D. Plaghki L. Mouraux A. (2011). The pain matrix reloaded: A salience detection system for the body. *Prog. Neurobiol.* 93 111–124. 10.1016/j.pneurobio.2010.10.005 21040755

[B28] Li K. Li R. Wang X. (2022). Research progress of music therapy to improve pain. *Chin. J. Rehabil. Med.* 37 112–116.

[B29] Lin S. Ye T. Jia N. (2009). Functional imaging of the brain in pain. *J. China Japan Friendsh. Hosp.* 23 301–303.

[B30] Luo L. Shan M. Zu Y. Chen Y. Bu L. Wang L. (2022). Effects of long-term COVID-19 confinement and music stimulation on mental state and brain activity of young people. *Neurosci. Lett.* 791:136922. 10.1016/j.neulet.2022.136922 36272556 PMC9580244

[B31] Martin-Saavedra J. S. Vergara-Mendez L. D. Talero-Gutierrez C. (2018). Music is an effective intervention for the management of pain: An umbrella review. *Complement. Ther. Clin. Pract.* 32 103–114. 10.1016/j.ctcp.2018.06.003 30057035

[B32] Mccracken L. M. Spertus I. L. Janeck A. S. Sinclair D. Wetzel T. F. (1999). Behavioral dimensions of adjustment in persons with chronic pain: Pain-related anxiety and acceptance. *Pain* 80 283–289. 10.1016/s0304-3959(98)00219-x 10204741

[B33] Meagher M. W. Albu S. (2016). Expectation of nocebo hyperalgesia affects EEG alpha-activity. *Int. J. Psychophysiol.* 109 147–152. 10.1016/j.ijpsycho.2016.08.009 27562424

[B34] Mobily P. R. Herr K. A. Kelley L. S. (1993). Cognitive-behavioral techniques to reduce pain: A validation study. *Int. J. Nurs. Stud.* 30 537–548. 10.1016/0020-7489(93)90025-p 8288423

[B35] Patrick D. W. (2000). *Textbook of pain.* Shenyang: Liaoning Education Press.

[B36] Peng K. Yucel M. A. Steele S. C. Bittner E. A. Aasted C. M. Hoeft M. A. (2018b). Morphine attenuates fnirs signal associated with painful stimuli in the medial frontopolar cortex (medial BA 10). *Front. Hum. Neurosci.* 12:394. 10.3389/fnhum.2018.00394 30349466 PMC6186992

[B37] Peng K. Yucel M. A. Aasted C. M. Steele S. C. Boas D. A. Borsook D. (2018a). Using prerecorded hemodynamic response functions in detecting prefrontal pain response: A functional near-infrared spectroscopy study. *Neurophotonics* 5:011018. 10.1117/1.NPh.5.1.011018 PMC5641587 29057285

[B38] Peretz I. (2001). “Listen to the brain: A biological perspective on musical emotions,” in *Music and emotion: Theory and research*, eds Juslin P. Sloboda J. (New York, NY: Oxford University Press), 105–134.

[B39] Pourshoghi A. Zakeri I. Pourrezaei K. (2016). Application of functional data analysis in classification and clustering of functional near-infrared spectroscopy signal in response to noxious stimuli. *J. Biomed. Opt.* 21:101411. 10.1117/1.jbo.21.10.101411 27155020

[B40] Qi Y. (2004). Related problems and clinical application of music therapy. *Chin. Nurs. Res.* 18, 473–474. 10.3969/j.issn.1009-6493.2004.06.002

[B41] Qin L. (2014). Research on ventromedial prefrontal cortex. *Guide Sci. Educ.* 11, 215–216. 10.16400/j.cnki.kjdkz.2014.04.050

[B42] Robb S. L. Burns D. S. Carpenter J. S. (2011). Reporting guidelines for music-based interventions. *Music Med.* 3 271–279. 10.1177/1943862111420539 23646227 PMC3641897

[B43] Schroeter M. L. Bucheler M. M. Muller K. Uludag K. Obrig H. Lohmann G. (2004). Towards a standard analysis for functional near-infrared imaging. *Neuroimage* 21 283–290. 10.1016/j.neuroimage.2003.09.054 14741666

[B44] Shao L. Wang T. (2009). Status and progress of music therapy. *Chin. J. Rehabil. Med.* 24 959–962.

[B45] Sharini H. Fooladi M. Masjoodi S. Jalalvandi M. Pour M. Y. (2019). Identification of the pain process by cold stimulation: Using dynamic causal modeling of effective connectivity in functional near-infrared spectroscopy (fNIRS). *Innov. Res. Biomed. Eng.* 40 86–94. 10.1016/j.irbm.2018.11.006 PMC7416094 32802795

[B46] Shi Z. Shi J. Liu C. (2022). Effects of exercise-psychology-sleep nursing intervention combined with mindfulness music intervention on fatigue degree, sleep quality and disease self-perceive burden in patients with glioma. *Clin. Res. Pract.* 7 151–153. 10.19347/j.cnki.2096-1413.202209041

[B47] Taesler P. Rose M. (2016). Prestimulus theta oscillations and connectivity modulate pain perception. *Journal Neurosci.* 36 5026–5033. 10.1523/jneurosci.3325-15.2016 27147655 PMC6601851

[B48] Tong H. Maloney T. C. Payne M. F. King C. D. Ting T. V. Kashikar-Zuck S. (2022). Processing of pain by the developing brain: Evidence of differences between adolescent and adult females. *Pain* 163 1777–1789. 10.1097/j.pain.0000000000002571 35297790 PMC9391252

[B49] Tracey I. Mantyh A. (2007). The cerebral signature for pain perception and its modulation. *Neuron* 55 377–391. 10.1016/j.neuron.2007.07.012 17678852

[B50] Usui C. Kirino E. Tanaka S. Inami R. Inoue R. (2020). Music intervention reduces persistent fibromyalgia pain and alters functional connectivity between the insula and default mode network. *Pain Med.* 21 1546–1552. 10.1093/pm/pnaa071 32330259

[B51] Vrana A. Meier M. L. Hotz-Boen De Rmaker S. Humphreys B. K. Scholkmann F. (2016). Different mechanosensory stimulations of the lower back elicit specific changes in hemodynamics and oxygenation in cortical sensorimotor areas-A fNIRS study. *Brain Behav.* 6:e00575. 10.1002/brb3.575 28031998 PMC5167005

[B52] Wang Y. Pan C. (2022). Etiological analysis and TCM treatment of myofascial pain syndrome. *Nei Mong. J. Tradit. Chin. Med.* 41 117–119. 10.16040/j.cnki.cn15-1101.2022.06.076

[B53] White J. M. (2000). State of the science of music interventions. *Crit. Care Nurs. Clin. N. Am.* 12 219–225. 10.1016/s0899-5885(18)30114-x 11249367

[B54] Xia W. Dai R. Xu X. Huai B. Bai Z. Zhang J. (2022). Cortical mapping of active and passive upper limb training in stroke patients and healthy people: A functional near-infrared spectroscopy study. *Brain Res.* 1788:147935. 10.1016/j.brainres.2022.147935 35500604

[B55] Xin J. Wang Y. Ao Q. Zuo H. (2012). Correlation between trigger pain and Oxy-Hb in the prefrontal cortex in trigeminal neuralgia with near-infrared spectroscopy. *Chin. J. Clin.* 6 5917–5921.

[B56] Yang C. L. Lim S. B. Peters S. Eng J. J. (2020). Cortical activation during shoulder and finger movements in healthy adults: A functional near-infrared spectroscopy (fNIRS) study. *Front. Hum. Neurosci.* 14:260. 10.3389/fnhum.2020.00260 32733221 PMC7362764

[B57] Ye Y. Xu Y. Yang Y. (2021). Clinical study on warm acupuncture and moxibustion for myofascitis of nape of wind-cold-dampness type. *J. N. Chin. Med.* 53 178–181. 10.13457/j.cnki.jncm.2021.21.042

[B58] Yennu A. Tian F. Gatchel R. J. Liu H. (2016). Prefrontal hemodynamic mapping by functional near-infrared spectroscopy in response to thermal stimulations over three body sites. *Neurophotonics* 3:045008. 10.1117/1.nph.3.4.045008 PMC5166717 28018934

[B59] Yucel M. A. Aasted C. M. Petkov M. P. Borsook D. Boas D. A. Becerra L. (2015). Specificity of hemodynamic brain responses to painful stimuli: A functional near-infrared spectroscopy study. *Sci. Rep.* 5:9469. 10.1038/srep09469 25820289 PMC4377554

[B60] Zhang L. Lv X. Hu L. (2021). Developmental cognitive neuroscience of pain: Present and future. *Sci. Sin.* 51 730–742.

[B61] Zhang W. Luo F. Han J. (2001). Progress in the study of pain by brain imaging. *Prog. Physiol. Sci.* 32 209–214. 12545791

[B62] Zheng W. Woo C. W. Yao Z. Goldstein P. Atlas L. Y. Roy M. (2020). Pain-evoked reorganization in functional brain networks. *Cereb. Cortex* 30 2804–2822. 10.1093/cercor/bhz276 31813959 PMC7197093

